# Targeting the PARylation-Dependent Ubiquitination Signaling Pathway for Cancer Therapies

**DOI:** 10.3390/biom15020237

**Published:** 2025-02-07

**Authors:** Daoyuan Huang, Jingchao Wang, Li Chen, Weiwei Jiang, Hiroyuki Inuzuka, David K. Simon, Wenyi Wei

**Affiliations:** 1 Department of Pathology, Beth Israel Deaconess Medical Center, Harvard Medical School, Boston, MA 02215, USA; 2 Department of Neurology, Beth Israel Deaconess Medical Center, Harvard Medical School, Boston, MA 02215, USA; dsimon1@bidmc.harvard.edu

**Keywords:** PARylation ubiquitination, RNF146, tankyrase

## Abstract

Poly(ADP-ribosyl)ation (PARylation) is a dynamic protein post-translational modification (PTM) mediated by ADP-ribosyltransferases (ARTs), which regulates a plethora of essential biological processes, such as DNA repair, gene expression, and signal transduction. Among these, PAR-dependent ubiquitination (PARdU) plays a pivotal role in tagging PARylated substrates for subsequent ubiquitination and degradation events through the coordinated action of enzymes, including the E3 ligase RNF146 and the ADP-ribosyltransferase tankyrase. Notably, this pathway has emerged as a key regulator of tumorigenesis, immune modulation, and cell death. This review elucidates the molecular mechanisms of the PARdU pathway, including the RNF146–tankyrase interaction, substrate specificity, and upstream regulatory pathways. It also highlights the biological functions of PARdU in DNA damage repair, signaling pathways, and metabolic regulation, with a focus on its therapeutic potential in cancer treatment. Strategies targeting PARdU, such as tankyrase and RNF146 inhibitors, synthetic lethality approaches, and immune checkpoint regulation, offer promising avenues for precision oncology. These developments underscore the potential of PARdU as a transformative therapeutic target in combating various types of human cancer.

## 1. Mechanism of Poly(ADP-Ribosyl)ation

**Figure 1:**
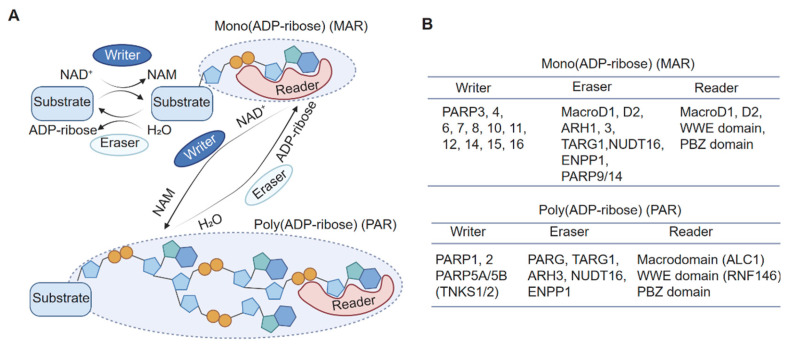
The protein ADP-ribosylation reaction and its working enzymes. (**A**) Protein ADP-ribosylation is dynamically regulated by writers (enzymes that add ADP-ribose) and erasers (enzymes that remove ADP-ribose) and recognized by readers (proteins or domains that bind ADP-ribose modifications). (**B**) Key writers, erasers, and readers in MARylation and PARylation.

Protein ADP-ribosylation is a reversible protein post-translational modification (PTM) in which ADP-ribose (ADPr) units are covalently transferred from nicotinamide adenine dinucleotide (NAD) to target molecules, releasing nicotinamide (NAM) as a byproduct [1,2,3]. This modification includes both mono-ADP-ribosylation (MAR) and poly-ADP-ribosylation (PAR) events and involves the coordinated actions of “writers” (enzymes that catalyze the modification), “erasers” (enzymes that remove it), and “readers” (proteins or domains that recognize and mediate downstream signaling), along with essential cofactors [4,5] (Figure 1A,B).

ADP-ribosylation is mediated primarily by the ADP-ribosyltransferase (ART) family of enzymes, which catalyze the attachment of ADPr units to specific amino acid residues such as serine (Ser), threonine (Thr), lysine (Lys), arginine (Arg), glutamate (Glu), aspartate (Asp), and cysteine (Cys) within the protein substrates [6,7,8,9]. Mono-ADP-ribosylating (MARylating) enzymes attach a single ADPr unit, while poly-ADP-ribosylating (PARylating) enzymes synthesize and attach PAR polymers. In humans, the ART family includes 17 known enzymes [2,10], with PARP1, PARP2, and tankyrases (TNKS1/2) catalyzing PARylation, while most others function primarily as MAR transferases [3].

Although proteins are the primary targets of ADP-ribosylation, recent studies have revealed its role in modifying DNA and RNA [11,12,13,14], thereby expanding its regulatory influence on critical biological processes such as DNA repair [6], gene expression [15], signal transduction [16,17], and energy metabolism [5,18]. This review focuses on the mechanisms, functions, and therapeutic potential of PAR-dependent ubiquitination.

## 2. Mechanism of PARylation-Dependent Ubiquitination

The understanding of PAR-dependent ubiquitination (PARdU) has significantly advanced in recent years. In 2009, researchers discovered that the tankyrase inhibitor XAV939 stabilizes Axin and suppresses Wnt signaling in part by blocking its degradation via the ubiquitin–proteasome pathway [19]. This process is largely mediated by tankyrase isoforms (TNKS1/2), which bind conserved domains in Axin for its PARylation, and the E3 ligase RNF146, which recognizes poly(ADP-ribose) (PAR) chains through its WWE domain to facilitate the subsequent ubiquitination and degradation of PARylated proteins [19,20,21,22].

Substrates of TNKS1/2, such as Axin1/2, rely on the conserved “tankyrase-binding motif” (TBM, RXXPDG) for PARdU [1,23,24]. Additional protein targets, including 3BP2 [25] and PTEN [16], have expanded the known substrate repertoire. Moreover, proteomic analyses have identified hundreds of potential PARdU substrates [20,26,27,28,29], emphasizing PARylation’s pivotal role in ubiquitination and protein degradation and providing a deeper understanding of its regulatory mechanisms.

### 2.1. Insights into RNF146 and Tankyrase Interactions

**Figure 2:**
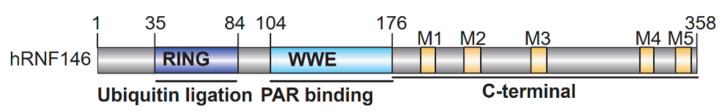
The protein domain features of human RNF146 E3 ubiquitin ligase. M1–M5 are putative TBMs.

RNF146 features an N-terminal WWE domain, which binds *iso*-ADP-ribose units within PAR chains, and a RING domain that mediates the ubiquitination process (Figure 2). Structural studies further reveal that PAR binding induces conformational changes in RNF146, activating its ubiquitin ligase activity [30]. This specificity distinguishes RNF146 from other E3 ubiquitin ligases and highlights its potential as a therapeutic target.

The C-terminal domain of RNF146 is critical for interacting with tankyrase (TNKS1/2). Five putative tankyrase-binding motifs (TBMs) have been identified within this domain, and at least four are essential for RNF146–tankyrase interactions [30,31] (Figure 2). The traditional TBM consensus sequence (RXXPDG) has been expanded to include broader variations such as RXX(A/G/P/V)XG or RXXX(A/G/P/V)XG [31], offering valuable insights into tankyrase-interacting proteins and potential PARdU substrates. This enhanced understanding provides a foundation for predicting and validating PARdU components in various cellular processes.

### 2.2. Mechanism of RNF146-Mediated Ubiquitination of PARylated Substrates

**Figure 3:**
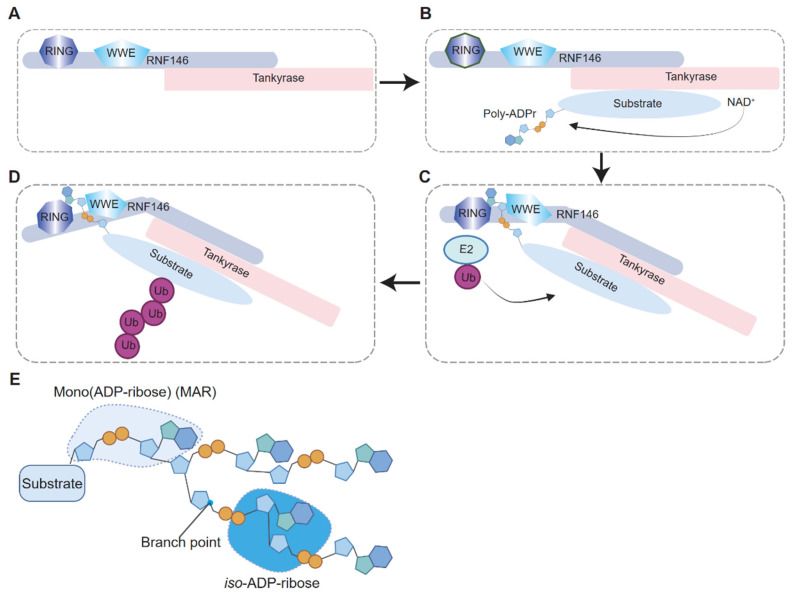
Molecular mechanism of RNF146 activation and substrate recognition in PAR-dependent ubiquitination. (**A**) RNF146 forms a protein complex with tankyrase via its C-terminal domain, while its RING domain remains in an inactive state, lacking E3 ubiquitin ligase activity. (**B**) Tankyrase catalyzes the poly(ADP-ribose) (PAR) modification of the downstream protein substrate. (**C**) The *iso*-ADP-ribose units within the PAR chain bind to the interface between the RING and WWE domains of RNF146, thereby inducing a conformational change in the RING domain that activates its E3 ubiquitin ligase activity. (**D**) The E3 ubiquitin ligase activity of the RNF146 RING domain is activated, enabling the transfer of ubiquitin to its substrate. (**E**) A schematic representation of the PAR chain on the substrate, with the *iso*-ADP-ribose unit highlighted in blue to indicate its critical role in RNF146 activation.

Studies have revealed that RNF146 exists in cells as part of a protein complex with tankyrase through its C-terminal domain [30]. Initially, the RING domain of RNF146 remains in an inactive state (Figure 3A). In this complex, tankyrase is responsible for substrate selection and PARylation. Upon PARylation, the PAR groups on the substrate bind to RNF146, thereby triggering a conformational switch that activates its E3 ligase activity [30] (Figure 3B,C). During this process, the PARylated substrate remains tethered within the RNF146–tankyrase complex through its interaction with tankyrase, providing the molecular basis for substrate ubiquitination in cells.

*Iso*-ADPr, the smallest internal structural unit of poly(ADP-ribose) (PAR), binds between the WWE and RING domains of RNF146 [30,32] (Figure 3D,E). This binding induces a conformational change in the RING domain, transitioning it from a catalytically inactive to an active state. In the absence of PAR, the RING domain cannot effectively bind to the E2 enzymes. To this end, the interaction between *iso*-ADPr and RNF146 enables the functional activation of the RING domain [30,32].

Since substrate PARylation and PARdU (PAR-dependent ubiquitination) are both catalyzed by enzymes within the same protein complex, substrate specificity in PARdU is likely governed primarily by substrate–tankyrase interactions. Maintaining the unliganded RNF146 in an inactive state may contribute to the stability of the RNF146–tankyrase complex, thereby regulating the steady-state activity of PARdU in cells.

## 3. Known Substrates of RNF146 and Their Biological Functions

The PAR-dependent ubiquitination (PARdU) mechanism, mediated by RNF146 and tankyrase, plays a pivotal role in various physiological and pathological processes [5,6]. This review comprehensively summarizes the known substrates of RNF146-mediated ubiquitination and the biological processes influenced by PARdU (Table 1). This mechanism involves the targeted ubiquitination of poly(ADP-ribosyl)ated substrates, playing a pivotal role in immune modulation, DNA repair, cell death regulation, neurodegeneration, and tumorigenesis. Through the precise regulation of substrate degradation, PARdU orchestrates a wide array of cellular functions and pathophysiological responses. Below, we provide a detailed overview of its important roles, organized into physiological and pathological contexts, with a specific focus on its regulatory mechanisms and potential therapeutic applications.

### 3.1. Physiological Functions of PARdU

In normal physiological contexts, PARdU plays an essential role in maintaining cellular homeostasis and regulating key signaling pathways. One prominent function is the regulation of epithelial lumen formation, where RNF146 facilitates the degradation of SH2BP5 and SH3BP5L, thereby inhibiting Rab11a activation [33]. This ensures proper morphogenesis and epithelial architecture. Additionally, PARdU stabilizes the Wnt/β-catenin signaling pathway through tankyrase-mediated poly(ADP-ribosyl)ation and RNF146-driven Axin1/2 degradation, a process critical for neurite outgrowth, synapse formation, and energy metabolism [18,34]. Another important role of PARdU is in cell cycle regulation, where it targets PARylated p21 for degradation [35], contributing to the progression of the cell cycle.

Proteomic studies have expanded our understanding of PARdU by identifying a diverse array of substrates. For instance, BLZF1 regulates gene transcription [20], while CASC3 disrupts spliceosome function [20,36]. Other notable substrates include OTUD5 [26], which influences de-ubiquitination processes [37], and PARP10 [26], a regulator of PARylation signaling [38]. Additionally, SARDH modulates sarcosine demethylation [26], showcasing PARdU’s impact on metabolic pathways. Further research has identified substrates such as Disc1, Striatin, Fat4, BCR, MERIT40, and RAD54 [25], emphasizing the complexity and breadth of PARdU-mediated cellular regulation.

### 3.2. Pathological Functions of PARdU

#### 3.2.1. Tumorigenesis and Immune Regulation

In pathological conditions, PARdU plays a significant role in tumor development and immune modulation. Furthermore, PARdU regulates tumorigenesis by targeting Axin1/2 for degradation, influencing the Wnt/β-catenin signaling pathway [19,20,39]. Additionally, PARdU-mediated degradation of AMOT family proteins regulates the YAP/TAZ pathway [17,40], and PTEN ubiquitination drives the activation of the PTEN-AKT signaling cascade [16], which accelerates tumor progression. It suppresses innate immunity by degrading MAVS [41] while also promoting tumor immune evasion through PARP1 degradation, which enhances STAT3 activity and upregulates PD-L1 expression [42]. In autoinflammatory diseases, tankyrase negatively regulates the homeostasis of 3BP2, also termed SH3 domain-binding protein 2, which functions as a signaling adaptor protein, through RNF146-mediated ubiquitination [25,27]. Mutations in 3BP2 disrupt this regulation [27,43], leading to Cherubism, an autosomal dominant disorder associated with craniofacial abnormalities [44].

#### 3.2.2. Parkinson’s Disease and PARthanatos

Neurodegenerative diseases, particularly Parkinson’s disease (PD), are another major area where PARdU exhibits regulatory functions [45]. Poly(ADP-ribose)-dependent cell death (PARthanatos) is characterized by the excessive synthesis of poly(ADP-ribose) (PAR) by PARP1 and is closely associated with neurodegenerative diseases and acute injuries such as stroke, MPTP-induced mitochondrial dysfunction, and NMDA-induced neuronal excitotoxicity [46,47,48]. Recently, PARthanatos has gained attention as a key focus in the study of progressive neurodegenerative diseases including Parkinson’s disease (PD) [48]. Notably, studies in PD mouse models have shown that PARthanatos is likely a primary driver of selective dopaminergic neuron loss during their gradual degeneration [49]. In PD, PAR is primarily synthesized by PARP1, although TNKS1/2-derived PAR may also contribute to neuronal PARthanatos. Furthermore, as we mentioned before, PAR synthesized by PARP1 and TNKS1/2 can mediate substrate ubiquitination through PAR-dependent ubiquitination (PARdU). This suggests that PARdU signaling could be a potential regulator of dopaminergic neuron loss in PD. To this end, studies have revealed that Crocetin, through interaction with the PD-related protein HK-I, inhibits RNF146-mediated degradation of PARylated HK-I, thereby preventing mitochondrial dysfunction and DNA damage during the later stages of PARthanatos and helping cells resist irreversible death [50]. Additionally, in SH-SY5Y cells, chlorogenic acid activates the Akt1-CREB-RNF146 signaling pathway, promoting RNF146 transcription [51]. RNF146, by binding PAR rather than relying on its E3 ubiquitin ligase activity, suppresses PARP1 activity, enhancing cell survival against 6-OHDA and α-synuclein aggregation [51]. In mice, chlorogenic acid similarly activates the Akt1-CREB-RNF146 signaling pathway in the brain, providing RNF146-dependent neuroprotection [51]. However, in post-mortem brain samples of PD patients, this pathway was found to be dysregulated [51]. These findings indicate that PARdU signaling plays an important regulatory role in PD pathogenesis. However, further investigation is needed to further elucidate its precise mechanisms and to develop PARdU-dependent therapeutic strategies for PD.

#### 3.2.3. DNA Damage Repair and Cell Death

PARdU also plays a pivotal role in DNA damage repair by targeting key proteins for degradation, including 53BP1 [52], CtIP [53], and BRD7 [54], which are essential for effective DNA damage responses and chemotherapy efficacy. In addition, it regulates cell death pathways, such as necroptosis via RIPK1 ubiquitination [55,56], oxidant-induced cell death through PARP1 degradation [57], and PARthanatos by targeting PARylated HK-I [50]. These functions underline PARdU’s significance in maintaining cellular integrity under stress conditions.

#### 3.2.4. Other Cellular Functions

Other notable functions include the regulation of cartilage matrix synthesis via SOX9 PARylation [58], which impacts osteoarthritis progression, and the degradation of p65 [59], which supports the efficacy of PARP inhibitors in BRCA wild-type cancer cells. Finally, PARdU-mediated degradation of PARP1 alleviates macrophage inflammation [60].

The majority of reported PARdU substrates undergo poly(ADP-ribosyl)ation mediated by tankyrase (TNKS1/2). However, the poly(ADP-ribosyl)ation of substrates such as p65, BRD7, PARP1, and CtIP is catalyzed by PARP1, suggesting that among the known PARylation writers (TNKS1/2, PARP1/2), only PARP2 has not yet been exactly reported to assemble the PARdU machinery to facilitate the PARdU process. However, the substrate-specific recognition mechanisms of TNKS1/2 and PARP1 remain to be elucidated and warrant further investigation.

**Figure 4:**
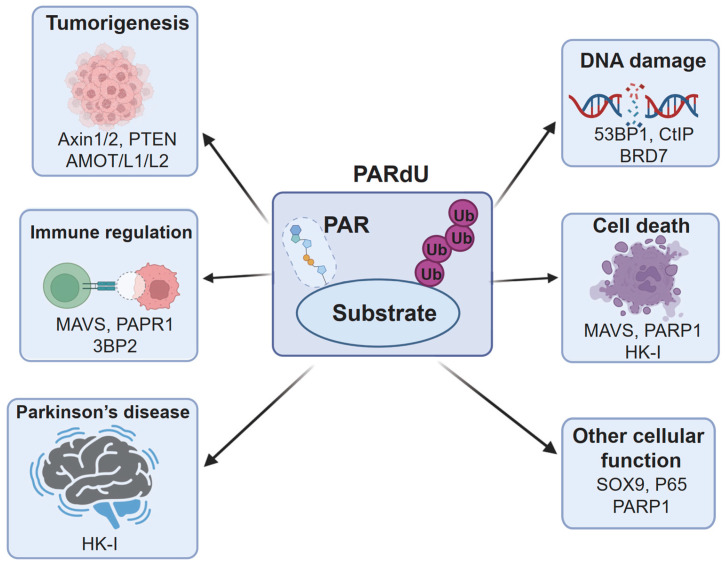
A schematic summary of PARdU substrates involved in various pathological signaling pathways.

By systematically analyzing PARdU substrates and their associated functions, we will gain a deeper understanding of their roles in physiological and pathological contexts, thereby providing a robust foundation for developing PARdU-based therapeutic strategies (Figure 4).

**Table 1:** Summary of known PARdU substrates and their functions.

PARylation Enzyme	Substrate	Regulation	Biological Pathway	Reference
Tankyrase	SH3BP5	Degradation	Regulates epithelial lumen formation	[33]
	SH3BP5L	Degradation	Regulates epithelial lumen formation	[33]
	3BP2	Degradation	Cherubism disease	[25,27,43]
	AXIN1/2	Degradation	Regulates neurite outgrowth	[34]
	AXIN1/2	Degradation	Regulates bone dynamics and energy metabolism	[18]
	AXIN1/2	Degradation	Wnt pathway	[19,20,39]
	RNF146	Degradation	E3 ligase	[20,27]
	p21	Degradation	Cell cycle	[35]
	LKB1	Block activation	Inhibits LKB1-AMPK signaling	[61]
	PTEN	Degradation	Promotes tumor growth	[16]
	FADD	Degradation	Inhibits TNF-induced cell death	[55]
	OTUD5	Degradation	De-ubiquitination	[26]
	PARP10	Degradation	PARylation	[26]
	SARDH	Degradation	Sarcosine demethylation	[26]
	AMOT/L1/L2	Degradation	Regulates YAP oncogenic activity	[17]
	Motins	Degradation	Regulates YAP/TAZ pathway	[40]
	MAVS	Degradation	Inhibits innate antiviral response	[41]
	53BP1	Degradation	DNA damage	[52]
	BLZF1	Degradation	Transcription factor	[20]
	SOX9	Degradation	Regulates osteoarthritic cartilage	[58]
	CASC3	Degradation	Spliceosome	[20,26]
TNKS1	RIPK1	Degradation	Block necroptosis	[56]
PARP1	p65	Degradation	Directs to combine bortezomib with niraparib for thyroid cancer	[59]
	BRD7	Degradation	DNA-damaging chemotherapeutic	[54]
	PARP1	Degradation	Regulates PD-L1 expression	[42]
	PAPR1	Degradation	Inhibits oxidant-induced cell death	[57]
	CtIP	Degradation	Homologous recombination repair	[53]
Undefined	PARP1	Degradation	Ameliorates macrophage inflammation	[60]
	HK-I	Degradation	Regulates PARthanatos	[50]

## 4. Targeting the PARdU Pathway for Cancer Therapy

Targeting the PARdU pathway represents a promising approach for cancer therapy, particularly in malignancies driven by dysregulated components such as TNKS1/2 and RNF146. Tankyrase inhibitors (e.g., XAV939, IWR-1, G007-LK) have shown potential by stabilizing substrates like Axin1/2 and suppressing hyperactive Wnt/β-catenin signaling [19,62], frequently observed in colorectal cancer and hepatocellular carcinoma [63,64,65,66]. However, the diverse substrates of tankyrase present challenges, necessitating the development of substrate-selective inhibitors to minimize off-target effects. Alternatively, directly targeting RNF146, the E3 ligase central to the PARdU pathway, offers another strategy. This can be achieved by inhibiting its WWE domain, which binds PAR, or its RING domain, which mediates ubiquitination. Although selective RNF146 inhibitors are currently lacking, efforts should focus on developing small molecules or peptides that disrupt WWE-PAR interactions or inhibit RING activity [67].

### 4.1. Regulating RNF146 to Enhance Therapeutic Strategies

Understanding the upstream regulatory signals of RNF146 is crucial for effectively targeting the PARdU pathway and identifying novel strategies for cancer therapy. Here, we summarize the currently known mechanisms regulating RNF146 expression and activity (Table 2).

In HCC, *RNF146* is identified as a novel HIF1α/2α target gene that is transcriptionally activated under hypoxic conditions. It promotes PTEN ubiquitination and degradation, thereby activating the AKT/mTOR signaling pathway, which drives HCC proliferation, clonogenicity, and glycolysis. Due to its critical role in HCC progression, RNF146 is considered a promising therapeutic target for anti-HCC strategies [68]. Additionally, Akt1, activated by chlorogenic acid, promotes CREB-dependent transcriptional activation of RNF146, which inhibits PARP1 by sequestering PAR rather than through its E3 ligase activity. This RNF146-mediated mechanism protects dopaminergic neurons from 6-OHDA toxicity and α-synuclein aggregation, highlighting its critical neuroprotective role in Parkinson’s disease [51]. Moreover, RNF146 is upregulated in the prefrontal cortex of valproic acid (VPA)-exposed mice, leading to dysregulation of the Wnt/β-catenin signaling pathway and impaired social behaviors. Overexpression of RNF146 in the prefrontal cortex enhances excitatory synaptic transmission, contributing to the social deficits observed in the VPA-induced ASD model [69]. However, the mechanism by which VPA exposure induces the upregulation of RNF146 protein remains unknown. Neuroprotectin D1 (NPD1) upregulates RNF146 (Iduna) expression, enhancing its poly(ADP-ribose) (PAR)-dependent activity to facilitate DNA repair and protect against cell death under oxidative stress. Systemic administration of DHA, the precursor of NPD1, increases Iduna levels in astrocytes and neurons, providing significant neuroprotection following ischemic stroke [70].

Post-translational modifications also play a critical role in regulating RNF146 E3 ligase activity. To this end, RNF146 undergoes SUMOylation primarily at lysine residues K19, K61, K174, and K175, with UBC9/PIAS3/MMS21 mediating SUMO3 conjugation and SENP1/2/6 facilitating its deconjugation. SUMOylation promotes RNF146 nuclear localization and enhances its interaction with Axin, accelerating Axin ubiquitination and degradation, thereby activating β-catenin signaling and driving HCC progression. Inhibiting RNF146 SUMOylation suppresses HCC progression, highlighting its SUMOylation as a potential therapeutic target [71]. Conversely, certain pathways negatively regulate RNF146. RNF146 is regulated by tumor-suppressive miRNAs miR-306 and miR-79, which target RNF146 mRNA, reducing its expression. This downregulation inhibits RNF146-mediated degradation of tankyrase, leading to hyperactivation of JNK signaling through a non-canonical pathway. While JNK activity is critical for tumor growth, excessive activation induced by miR-306 and miR-79 drives tumor elimination, highlighting RNF146 as a key modulator in JNK signaling and a potential target for miRNA-based cancer therapies [72]. Moreover, *RNF146* transcription is suppressed by RANKL through an NF-κB-related inhibitory element in its promoter, stabilizing its substrates 3BP2 and AXIN1. This leads to SRC activation and β-catenin downregulation, essential for osteoclastogenesis. RNF146 acts as a regulatory switch to control osteoclast differentiation and cytokine production, implicating it as a potential target in chronic inflammatory diseases [73]. The regulation of protein homeostasis through ubiquitination and de-ubiquitination presents a promising therapeutic avenue for cancer treatment [74]. RNF146 mediates PARdU-dependent degradation of key tumor suppressors such as AXIN1/2 and PTEN, thereby promoting tumorigenesis. Hence, investigating the regulatory mechanisms of RNF146 protein homeostasis, particularly its deubiquitinases, may offer new strategies for therapeutic intervention. Targeting RNF146-specific deubiquitinase inhibitors to lower RNF146 protein levels could be an effective treatment for HCC and other Wnt-dependent cancers.

**Figure 5:**
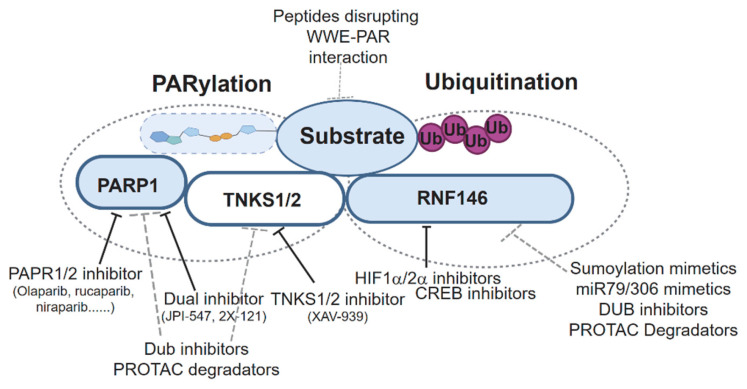
Strategies for targeting the PARdU signaling pathway for cancer treatments.

These insights suggest opportunities for therapeutic interventions, including the use of HIF1α/2α inhibitors [75,76] and CREB inhibitors [77], the development of SUMOylation mimetics and *miRNA mimics*, and the identification of potential RNF146-specific deubiquitinase (DUB) inhibitors to suppress RNF146 pathways, thereby improving therapeutic outcomes (Figure 5).

### 4.2. Restoring Tumor Suppressors and Enhancing Combination Therapies

Restoring the stability of tumor suppressors degraded by PARdU, such as PTEN, AMOT family proteins, and Motins, presents a promising strategy to bolster cancer treatments. This can be achieved through deubiquitinase (DUB) activators, which counteract protein ubiquitination induced by RNF146 activity, or proteasome inhibitors, which block substrate degradation. These approaches are particularly relevant in cancers with PTEN-AKT pathway dysregulation or YAP/TAZ activation.

Since PARP1 and tankyrase are key writers of the PARdU pathway, their inhibitors have been a focus of therapeutic development. A comprehensive summary of PARP1 and tankyrase inhibitors [78], including those approved for clinical use or undergoing clinical trials, is provided in Table 3. PARP1/2 inhibitors function primarily through two mechanisms: enzymatic inhibition and PARP1 trapping [79,80]. By blocking PARP1 activity, they prevent single-strand break (SSB) repair, while PARP1 trapping, achieved by mimicking NAD⁺ and competitively binding to PARP1’s catalytic domain, enhances its affinity for DNA damage sites [81,82]. This trapping obstructs DNA repair protein recruitment and results in replication-associated double-strand breaks (DSBs). In homologous recombination-deficient (HRD) tumors, such as BRCA1/2-mutant cancers, this synthetic lethality induces tumor cell death while sparing normal cells. Dual-target PARP-TNKS inhibitors, like 2X-121/JPI-547, combine PARP1/2 inhibition with tankyrase (PARP5a/b) suppression, disrupting DNA repair and Wnt/β-catenin signaling [83,84]. While this dual approach enhances anti-cancer efficacy, it also poses a higher risk of toxicity due to tankyrase’s diverse cellular roles. These inhibitors represent promising strategies for targeting HRD tumors and pathways critical for tumor survival. These inhibitors have demonstrated efficacy in targeting key pathways regulated by PARdU signaling. As writers of the PARdU pathway, tankyrases regulate critical processes such as Wnt/β-catenin signaling [19,20,39] and adherens junction (AJ) assembly [85], both of which play central roles in tumorigenesis. Tankyrase inhibitors, such as XAV939, RK-582, and LZZ-02 (binding the nicotinamide subsite, NS) [86,87,88] and G007-LK and K-756 (targeting the adenosine subsite, AS) [89,90], have shown therapeutic potential. For instance, these inhibitors stabilize PARylated substrates like Axin1/2, suppressing hyperactive Wnt signaling, a hallmark of APC-mutant cancers. Furthermore, tankyrase inhibition disrupts AJ assembly by preventing F-actin anchoring to cadherins, a process associated with cancer cell adhesion and metastasis. Combining tankyrase inhibitors with other targeted therapies, such as PI3K inhibitors (e.g., BKM120) and EGFR inhibitors (e.g., erlotinib), has been proposed as a promising approach for treating APC-mutant colorectal cancers [91,92]. These strategies can restore tumor suppressor stability, inhibit oncogenic signaling, and disrupt key mechanisms of cancer progression.

Tankyrase inhibition, coupled with emerging insights into PARdU signaling, represents a cornerstone of precision oncology, offering transformative potential in cancer therapy.

### 4.3. Targeting the Tankyrase-Binding Motif (TBM) and the PARdU Pathway

Tankyrase-binding motifs (TBMs) in PARdU substrates present another therapeutic target. Small molecules or mimetics that competitively inhibit TBM binding to tankyrase could prevent substrate PARylation and degradation. Furthermore, combining PARdU inhibitors with targeted therapies, such as Wnt pathway inhibitors [93], PI3K/AKT inhibitors [93,94], or immunotherapies [95], may improve efficacy and overcome resistance.

### 4.4. Exploiting PARdU in DNA Damage Repair and Cell Death Regulation

The PARdU pathway is critical in DNA damage repair, with RNF146 mediating the degradation of proteins like 53BP1 and BRD7, which are key to DNA damage response pathways. Modulating this process could sensitize cancer cells to DNA-damaging agents, enhancing the efficacy of chemotherapies or radiotherapy [29,54,96,97]. Similarly, PARdU regulates cell death pathways by targeting proteins such as RIPK1 (necroptosis), PARP1 (oxidant-induced cell death), and HK-I (PARthanatos). Hence, inhibiting PARdU could amplify pro-apoptotic signals or disrupt pro-survival mechanisms in cancer cells [55,98].

### 4.5. Future Directions and Drug Development

Given the complexity of PARdU signaling and potential resistance to monotherapies, targeting the PARdU pathway offers a multifaceted strategy to mitigate such resistance by intersecting multiple signaling networks. This could be achieved through the following possible approaches: developing proteolysis-targeting chimeras (PROTACs) for selective degradation of PARdU components and their positive regulators (Figure 5); conducting proteomics and CRISPR screens to identify new PARdU substrates and their interactions; designing *iso*-ADPr mimetics to disrupt RNF146-substrate interactions; and combining PARdU inhibitors with immune checkpoint blockade therapies to modulate PD-L1 degradation and enhance anti-tumor immunity.

By balancing specificity, efficacy, and safety, PARdU modulation has the potential to revolutionize cancer therapy. Integrating pathway-specific inhibitors, synthetic lethality strategies, and precision medicine approaches will be essential to fully harness the therapeutic potential of this pathway.

## 5. Conclusions: The Complexity of the PARdU Signaling Pathway

The poly(ADP-ribose)-dependent ubiquitination (PARdU) mechanism highlights a multifaceted regulatory system where poly(ADP-ribose) (PAR) synthesis and interaction with specific E3 ubiquitin ligases, such as RNF146, play critical roles. However, the complexity of this system raises several open questions and avenues for further exploration. First, while RNF146’s activation through its WWE domain binding to iso-ADPr has been well documented [20,32,99,100], the potential involvement of other PARP enzymes beyond PARP5a/b in synthesizing PAR modifications for E3 ligase activation remains less clear. For instance, PARP1 and PARP2 are suggested to contribute to PARdU, as evidenced by their interactions with RNF146 [2,101,102], while the diverse subcellular localizations of different PARPs may add specificity to the substrates targeted for degradation [103].

Furthermore, the possibility of other E3 ubiquitin ligases with WWE domains, such as HUWE1, TRIP12, or DTX1-4 [104], participating in PARdU suggests a broader spectrum of mechanisms beyond RNF146 [32]. These ligases, with their unique catalytic mechanisms (RING or HECT), may exhibit distinct modes of activation and substrate specificity. Whether PAR binding is essential for their activation or simply facilitates proximity to substrates remains an open question, as does the role of non-WWE domain-containing ligases in PARdU.

Another layer of complexity lies in the biochemical properties of PAR itself. PAR may function as a direct scaffold for the E3 ligase–substrate interaction, as seen in RNF146’s PARP1-mediated ubiquitination [100], or act indirectly by stabilizing E3–substrate complexes. The observation that free PAR polymers can activate RNF146 and promote auto-ubiquitination introduces the intriguing possibility that free PAR molecules, rather than PARylated substrates, may serve as key regulators in specific contexts [100].

Finally, emerging evidence suggests that PARdU is not a monolithic process but instead a dynamic and context-dependent mechanism. For example, PARP-mediated mono-ADP-ribosylation (MARylation) could serve as a precursor for PAR synthesis, with subsequent elongation catalyzed by PARP5a/b or other PARPs [5]. This sequential process raises questions about the interplay between MARylation and PARylation in controlling E3 ligase activity.

In summary, the complexity of PARdU reflects its integration of diverse molecular players and regulatory mechanisms, with spatial, temporal, and substrate-specific factors adding layers of control. Future studies should aim to unravel the interplay between different PARPs, E3 ligases, and free PAR molecules, which could provide deeper insights into PARdU’s physiological role in cellular homeostasis and disease and guide the development of novel therapeutic strategies targeting this intricate system.

## Figures and Tables

**Table 2:** Summary of the upstream regulators of RNF146.

Regulator	Positive	Negative	Function	Reference
HIF1α/2α	+	−	Promotes *RNF146* transcription	[68]
Chlorogenic acid	+	−	Promotes *RNF146* transcription	[51]
Valproic acid exposed	+	−	Upregulates RNF146 protein	[69]
NPD1	+	−	Upregulates RNF146 protein	[70]
Sumo3	+	−	Accelerates Axin degradation	[71]
miR-79/306	−	+	Downregulates RNF146	[72]
RANKL	−	+	Suppresses *RNF146* transcription	[73]

**Table 3:** Overview of PARP1/2 and tankyrase inhibitors approved for clinical application and undergoing clinical trials [78].

Inhibitor	Indication	Targets
Olaparib	Recurrent ovarian cancer, BRCA mutation	PARP1/2
Rucaparib	BRCA-mutated ovarian cancer Recurrent or relapsed ovarian cancer	PARP1/2
Niraparib	Ovarian cancer without germ-line or somatic mutation	PARP1/2
Talazoparib	Germline BRCA mutations, EGF2-negative, locally advanced or metastatic breast cancer	PARP1/2
Fluzoparib	Platinum-sensitive recurrent ovarian cancer Fallopian tube cancer Primary peritoneal cancer with germline BRCA mutation	PARP1/2
Pamiparib	Germline BRCA mutation-associated Recurrent advanced ovarian Fallopian tube or primary peritoneal cancer	PARP1/2
CVL218	Advanced solid tumor	PARP1/2
RP12146	Solid tumor Extensive-stage small-cell lung cancer Locally advanced breast cancer	PARP1/2
IDX-1197	Solid tumors; HR repair gene Mutation or deficiency gastric cancer	PARP1/2
Senaparib	Solid tumor Advanced solid tumors	PARP1/2
AMXI-5001	Advanced malignant neoplasm Breast cancer Ovarian cancer	PARP1/2
AZD5305	Breast cancer Ovarian cancer Pancreatic cancer	PARP1
AZD9574	Advanced solid malignancies	PARP1
JPI-547	Ovarian cancer pancreatic ducal Adenocarcinoma	PARP1/2 TNKS1/2
2X-121	Metastatic breast cancer	PARP1/2 TNKS1/2

## Data Availability

No new data were created or analyzed in this study.
